# Use of wild edible and nutraceutical plants in Raya-Azebo District of Tigray Region, northern Ethiopia

**DOI:** 10.1186/s41182-023-00550-8

**Published:** 2023-10-24

**Authors:** Mirutse Giday, Tilahun Teklehaymanot

**Affiliations:** Aklilu Lemma Institute of Pathobiology, Addis Ababa University, P.O. Box 1176, Addis Ababa, Ethiopia

**Keywords:** Wild edible plants, Nutraceutical plants, Raya-Azebo, Tigray, Ethiopia

## Abstract

**Background:**

Although there is a wide use of wild edible plants (WEPs) in Ethiopia, very little work has so far been done, particularly, in the Tigray Region, northern Ethiopia, to properly document the associated knowledge. The purpose of this study was, therefore, to document knowledge and analyze data related to the use of wild edible and nutraceutical plants in Raya-Azebo District of Tigray Region. The district was prioritized for the study to avoid the further loss of local knowledge and discontinuation of the associated practices because of the depletion of wild edible plants in the area mainly due to agricultural expansion and largely by private investors.

**Methods:**

A cross-sectional ethnobotanical study was carried out in the study District to collect data through individual interviews held with purposively selected informants, observation, market surveys, and ranking exercises. Descriptive and inferential statistical methods were employed to analyze and summarize the data using Statistical Package for Social Sciences (SPSS) version 16.

**Results:**

The study documented 59 WEPs, the majority of which (57.63%) were sought for their fruits. Most of the WEPs (49 species) were consumed in the autumn, locally called qewei, which includes the months of September, October, and November. *Ziziphus spina-christi* L. Desf., *Balanites aegyptiaca* (L.) Del. and *Opuntia ficus-indica* (L.) Miller were the most preferred WEPs. Both interviews and local market surveys revealed the marketability of *Opuntia ficus-indica, Ziziphus spina-christi, Ficus vasta* Forssk.*, Ficus sur* Forssk.*,* and *Balanites aegyptiaca*. Of the total WEPs, 21 were reported to have medicinal (nutraceutical) values, of which *Balanites aegyptiaca* and *Acacia etbaica* scored the highest rank order priority (ROP) values for their uses to treat anthrax and skin infections, respectively.

**Conclusions:**

The current investigation demonstrated the wide use of WEPs in the district. In future nutritional composition analysis studies, priority should be given to the most popular WEPs, and nutraceutical plants with the highest ROP values.

## Background

Wild edible plants (WEPs) play an important role in the livelihood of many rural communities across the world, particularly, in providing reliable alternatives when the production of cultivated crops decreases or fails [1–5]. Wild edible plants serve as source of vitamins, carbohydrates, proteins, fibers and minerals and are particularly rich in vitamins A and C, zinc, iron, calcium, iodine, thiamine, riboflavin, niacin, and folacin. Moreover, WEPs are valuable for the development of new food crops through domestication and in serving as a genetic resource pool needed to improve the productivity of cultivars [5, 6]. They provide a good source of cash income for local communities in different parts of the world [7–9]. There is also a long history of use of WEPs by communities in different parts of the world as medicines (nutraceuticals) to manage various ailments [10, 11], and reports show that such plants are still serving as an important source of medicines in the prevention and treatment of diseases [12, 13].

There is a wide use of WEPs in Ethiopia as supplement foods as revealed by different ethnobotanical studies [14–24]. Furthermore, studies show the utilization of WEPs in the country as nutraceuticals [25–27]. However, very little work that covered very limited geographical area has so far been done in Tigray Region, northern Ethiopia, to document local knowledge related to the use of WEPs [28–31]. A study conducted in Indaselassie-Shire District (North Western Tigray Zone) documented eight wild and semi-wild edible plants [29]. A survey carried out in Laelay Maichew and Tahtay Maichew districts (Central Tigray Zone) reported the use of three WEPs [28]. A study conducted in Raya-Alamata district (Southern Tigray Zone) revealed the use of 37 wild and semi-wild edible plants [30]. Another study carried out in Kilte Awlaelo district (Eastern Tigray Zone) recorded the use of 30 wild and semi-wild edible plants [31]. To the knowledge of the authors, there is no report of previous conduct of ethnobotanical study in Raya-Azebo district that aimed at documenting the use of WEPs. The purpose of this study was, therefore, to document and analyze ethnobotanical data mainly related to the use of wild edible and nutraceutical plants in Raya-Azebo District in the Southern Zone of the Tigray Region, northern Ethiopia. Raya-Azebo District was prioritized for the study because of an ongoing decimation of WEPs in the area due to destruction of their natural habitats attributed to mainly expansion of agriculture [32] and largely by private investors, which in the absence of proper and immediate documentation could ultimately bring about the perpetual loss of the local knowledge and practices associated with the use of WEPs.

## Methods

### The study area

Raya-Azebo District belongs to the Southern zone of the Tigray Region in northern Ethiopia and is located at latitudes between 12^o^ 15’and 13^o^ 41’ North and longitudes between 38^o^ 59’and 39^o^ 54’ East [33]. Raya-Azebo covers an area of about176, 210 ha [34]. The district is divided into 18 rural and two urban tabiyas (sub-districts) [35], and has a human population of 135, 870, of which 67,687 are men and 68,183 are women [36]. Ninety percent of the total area in the district is midland (1500–2300 m above sea level) while 10% is lowland (< 1500 m above sea level) [34]. The district gets its main rainfall between July and September and light rainfall between February and April. Agriculture is the main economic stay in the district. Sorghum and maize are the crops that are widely cultivated in the area. Malaria is the leading disease in the district causing high morbidity (unpublished data, Raya-Azebo District Health Office, 2015).

### Selection of study areas and informants

**Fig. 1:**
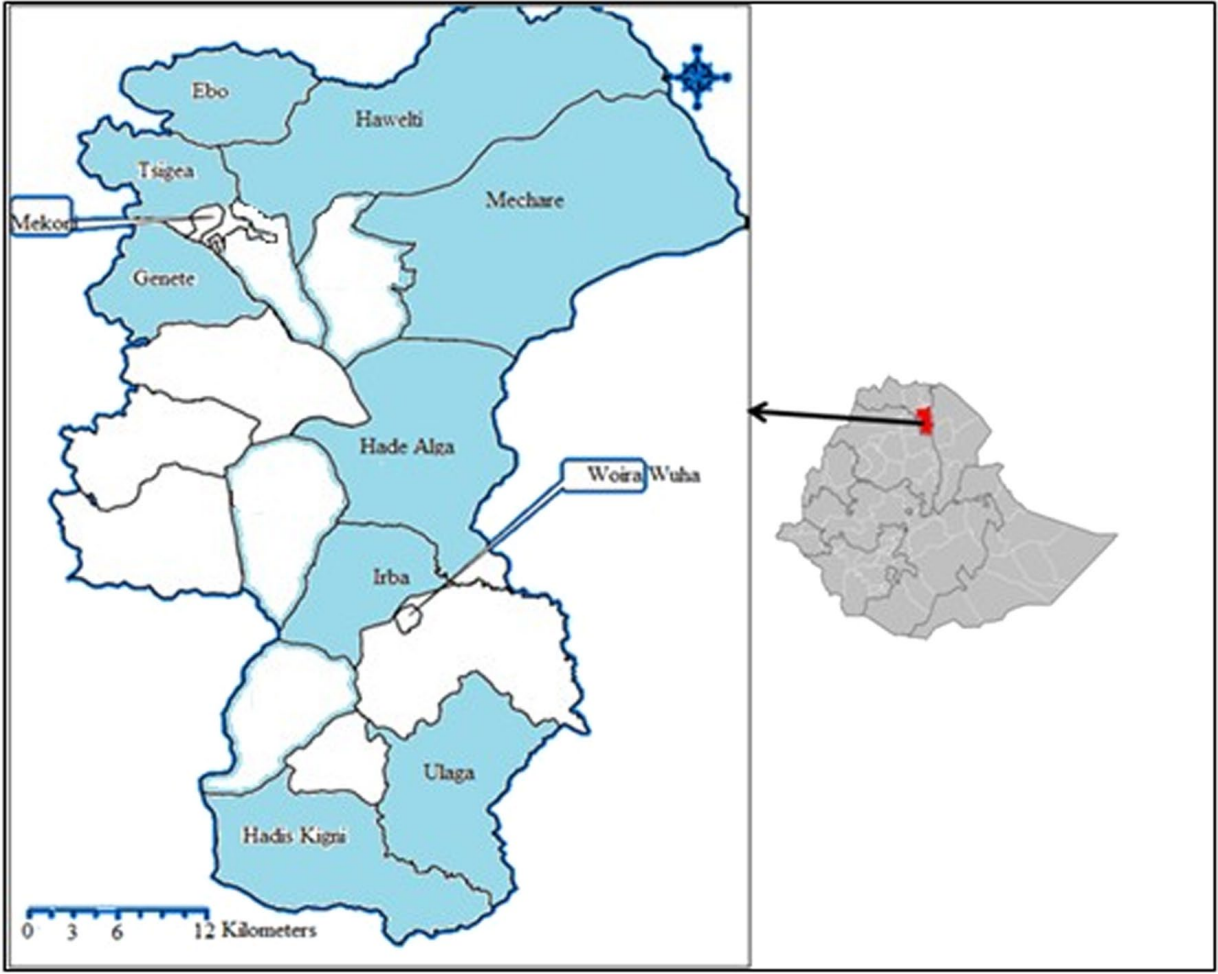
Map of Raya-Azebo District in Tigray Region of Ethiopia with selected study tabiyas in blue color

For the study, nine tabiyas that were relatively considered to have better vegetation cover and availability of knowledgeable individuals concerning use of WEPs were purposively sampled out of the total 18 rural tabiyas of the district with the help of experts at Raya-Azebo District Agriculture and Natural Resources Conservation Office. The selected tabiyas included Ebo, Erba, Genete, Hade Alga, Hadis Kigni, Hawelti, Mechare, Tsigea and Ulaga (Fig. 1). For the interview survey, a total of 180 informants constituting 158 men and 22 women aged 20 years and above were involved; 20 informants from each of the nine sampled tabiyas that were considered the most knowledgeable with regard to use of wild edible and nutraceutical plants were purposively identified and sampled with the help of tabiya administrators and elders.

### Methods of data collection

A cross-sectional survey was conducted in the study District between July 2017 and October 2018 and ethnobotanical data were collected through individual interviews that were held with the purposively selected informants using a pre-tested list of interview items (semi-structured questionnaire), field observation and market surveys following the methods stated in Martin [37]. Attempt was made to make the data collection process valid and reliable through the strict of use of pre-tested. Data collected mainly included local name of each claimed edible plant, edible part, maturity level at the time of collection, month of harvest, processing method, taste, habitat, availability status and potential threats. Additional data were collected concerning the medicinal (nutraceutical) value of each claimed edible plant. Voucher specimens were collected for most of the claimed WEPs plants and identified, and duplicates were deposited at the National Herbarium of the Addis Ababa University (AAU) and the mini-herbarium of the Aklilu Lemma Institute of Pathobiology (ALIPB), AAU.

### Data analysis

Microsoft Excel version 2016 was employed to enter and organize the data. Descriptive statistical methods were used to analyze and summarize the data using Statistical Package for Social Sciences (SPSS) version 16. Comparison of mean differences between informant groups was made using one-way analysis of variance (ANOVA) and differences in means with *p*-value ≤ 0.05 were considered statistically significant. Mean values are presented as mean plus or minus standard error of the mean (mean ± SEM). Preference ranking exercises were performed on WEPs of the highest informant consensus by involving individuals randomly sampled from the list of informants who participated in interviews following the method of Martin [37]. Preference ranking exercises were additionally conducted by the same randomly sampled individuals to identify main factors responsible for the depletion of WEPs. The relative healing potential of each nutraceutical plant cited by three or more informants for its use to manage a specific ailment was estimated by using an index called Fidelity Level (FL) with the formula FL = Ip/Iu × 100, where Ip/Iu × 100, where Ip is the number of informants who reported the utilization of the nutraceutical plant against a specific ailment and Iu is the total number of informants who mentioned the use of same plant against any ailment [38]. However, plants with similar FL values but known to different numbers of informants may differ in their healing potential. To differentiate the healing potential of plants of similar FL values, there is a need to calculate a correlation index known as relative popularity level (RPL) and determine rank order priority (ROP) value by multiplying FL value by RPL value [38]. RPL values range between 0 and 1. Plants are categorized into “popular” (RPL = 1) and “unpopular” (RPL < 1) groups. Popular plant are those cited by half or more of the highest number of informants (29 in the current study) who cited a given plant against any ailment. Accordingly, a medicinal plant cited by 15 or more of informants for its use against any ailment in the study District was considered popular and was assigned with an RPL value of 1, whereas a medicinal plant that was mentioned by less than 15 informants for its use against any ailment was considered unpopular and was assigned with RPL value less than 1 and was determined by dividing the total number of informants who mentioned the given plant against any ailment by 15.

## Results

### Diversity of wild edible plants

The study documented a total of 59 WEPs, of which 51 (belonging to 33 families and 40 genera) were, at least, identified to a genus level (44 to a species level and seven to genus level). The remaining eight species were only known by their Tigrigna names, as informants were not willing to travel to far distances to collect their specimens for identification purpose (Table 1). The families Asclepiadaceae, Fabaceae and Tiliaceae were represented by four species each, and the families Brassicaceae and Moraceae were represented by three species each. The families Anacardiaceae, Boraginaceae, Flacourtiaceae, Polygonaceae, Rhamnaceae and Rosaceae were represented by two species each, while the remaining 21 species were represented by a single species each. Of all the 40 genera recorded, the genus *Grewia* contributed four species, the genera *Acacia* and *Ficus* contributed three species each, and the genera *Rhus*, *Cordia*, *Brassica*, *Dovyalis* and *Rumex* contributed two species each, while the remaining 31 genera were represented by one species each. Of the plants that were determined, at least, to a genus level, 18(35%) were shrubs, 18 (35%) were herbs and 15 (29%) were trees.

**Table 1:** Wild edible plants consumed in Raya-Azebo District

Plant species name	Family name	Growth habit	Plant local name	Part consumed	Mode of preparation and consumption	No. of informant reports	Voucher no.
*Acacia abyssinica* Hochst. ex Benth	Fabaceae	Tree	Chea	Gum	Gum chewed and juice swallowed	1	MT-034
*Acacia etbaica* Schweinf	Fabaceae	Tree	Kariwora	Gum	Gum chewed and juice swallowed	2	MT-076
*Acacia seyal* Del	Fabaceae	Tree	Wacho	Gum	Gum chewed and juice swallowed	1	MT-003
*Amaranthus hybridus* L	Amaranthaceae	Herb	Hamlitilian	Seed	Seeds ground and eaten after baking	10	MT-017
				Leaf	Leaves chopped, boiled and eaten after decanting liquid and adding salt, paper, and powdered linseed		
*Balanites aegyptiaca* (L.) Del	Balanitaceae	Tree	Bedano	Fruit	Fruit eaten with or without the skin	134	MT-146
*Brassica nigra* (L.) Koch	Brassicaceae	Herb	Hamlisenafich	Leaf	Leaves boiled and eaten after decanting liquid and adding salt, pepper and powdered linseed	1	MT-019
*Brassica rapa* L	Brassicaceae	Herb	Hamli	Leaf	Leaves boiled and eaten with injera after decanting liquid and adding pepper and salt	1	MT-021
*Capsella bursa-pastoris* (L.) Medic	Brassicaceae	Herb	Hamliuf	Leaf	Leaves boiled and eaten with injera after decanting water	1	MT-015
*Carissa spinarum* L	Apocynaceae	Shrub	Agam	Fruit	Fruit eaten with or without the skin	96	MT-107, MT-157
*Celtis africana* Burm. f	Ulmaceae	Tree	Tselim om	Fruit	Fruit eaten	1	MT-008
*Cleome gynandra* L	Capparaceae	Herb	Abetiye	Leaf	Leaves boiled and eaten with injera after decanting liquid and adding butter and pepper	42	MT-047
*Commelina* sp.	Commelinaceae	Herb	Meanqor	Leaf	Eaten it is with injera	1	MT-198
*Commiphora africana* (A. Rich.) Engl	Burseraceae	Tree	Anqua	Root	Root chewed and juice swallowed	2	MT-020
*Cordia africana* Lam	Boraginaceae	Tree	Awhi	Fruit	Fruit chewed and swallowed without the stone	1	MT-069
*Cordia monoica* Roxb	Boraginaceae	Shrub	Maitero	Fruit	Fruit chewed and swallowed without seed	25	MT-142
*Cynanchum abyssinicum* Decne	Asclepiadaceae	Herb	Asemo	Root	Root chewed and juice swallowed	81	MT-133, MT-134
			Hamliasemo	Leaf	Leaves chopped and eaten with injera after decanting liquid and adding pepper and powdered linseed		
*Cyphostemma* sp.	Vitaceae	Herb	Tiwlahmi	Fruit	Fruit eaten	2	MT-038
*Diospyros mespiliformis* Hochst. ex A. DC	Ebenaceae	Tree	Yalue	Fruit	Fruit eaten	9	MT-005
*Dobera glabra* (Forssk.) Pair	Salvadoraceae	Shrub	Garsa	Fruit	Fruits boiled and eaten	10	MT-018
				Leaf	Boiled leaves eaten after decanting liquid and adding salt and pepper		
				Root	Root chewed and juice swallowed		
*Dovyalis abyssinica* (A.Rich.) Warb	Flacourtiaceae	Tree	Mengolhats	Fruit	Fruit eaten without the skin	10	MT-024
*Dovyalis verrucosa* (Hochst.) Warb	Flacourtiaceae	Shrub	Tiumtegna	Fruit	Fruit eaten	4	MT-006
*Echidnopsis* sp.	Asclepiadaceae	Herb	Dula	Leaf	Leaves eaten	6	MT-131
				Stem	Stem chewed and juice swallowed		
				Fruit	Fruit eaten		
*Eragrostis* sp.	Poaceae	Herb	Taftafo	Seed	Ground seeds are eaten after baking	2	MT-108
*Ficus carica* L	Moraceae	Tree	Beles	Fruit	Fruit eaten after peeling off the skin	1	MT-028
*Ficus sur* Forssk	Moraceae	Tree	Shamfa	Fruit	Fruit eaten after rubbing off the inside part and peeling off the skin	20	MT-027
*Ficus vasta* Forssk	Moraceae	Tree	Daero	Fruit	Fruit eaten after rubbing off the inside part and peeling off the skin	6	MT-030
*Grewia bicolor* Juss	Tiliaceae	Shrub	Habile	Fruit	Fruit chewed after removing skin and juice swallowed	3	MT-013
*Grewia mollis* A.Juss	Tiliaceae	Shrub	Reway	Fruit	Fruit chewed and swallowed after spitting seeds	12	MT-081
*Grewia* sp.	Tiliaceae	Shrub	Dianka	Fruit	Fruit chewed and swallowed after spitting seeds	69	MT-077, MT-138
*Grewia villosa* Willd	Tiliaceae	Shrub	Agewde	Fruit	Fruit chewed, juice swallowed and seeds spit	45	MT-054, MT-079
*Huernia macrocarpa* (A. Rich.) Sprenger	Asclepiadaceae	Herb (succulent)	Hamashiro	Aboveground	Leaves eaten after adding salt	29	MT-014
*Myrsine africana* L	Myrsinaceae	Shrub	Qachemo	Fruit	As it is	1	MT-007
*Olea europaea* subsp. *cuspidata* (Wall. ex G.Don) cif	Oleaceae	Tree	Awlie	Stem (bark)	Bark pounded and is added to tej (local honey drink) for good flavor	2	MT-174
				Leaf	Leaves boiled in water and tea drunk		
*Opuntia ficus-indica* (L.) Miller	Cactaceae	Shrub	Qolahri/beles	Fruit	Fruit eaten after peeling off the skin	121	MT-009
*Oxalis* sp.	Oxalidaceae	Herb	Chew chewa (chew mirakuit)	Above ground	Aboveground eaten	5	MT-041
*Pappea capensis* Eckl. & Zeyh	Sapindaceae	Tree	Tantaso	Fruit	Fruit eaten without seed	2	MT-051
*Pelargonium* sp.	Geraniaceae	Herb	Chewchewa	Leaf	Leaves eaten	1	MT-088
*Pentarrhinum insipidum* E.Mey	Asclepiadaceae	Herb	Gumgumo	Fruit	Fruit chewed after peeling off skin and juice swallowed	8	MT-121
*Rhus glutinosa* A.Rich	Anacardiaceae	Shrub	Tetaelo	Fruit	Fruit eaten	4	MT-023
*Rhus natalensis* Krauss	Anacardiaceae	Shrub	Atami	Fruit	Fruit chewed after peeling off skin and juice swallowed	20	MT-037, MT-097
*Rosa abyssinica* Lindley	Rosaceae	Shrub	Qaqa (chaga)	Fruit	Fruit chewed after peeling off skin and juice swallowed without seeds	4	MT-026
*Rubus steudneri* Schweinf	Rosaceae	Shrub	Mengolel	Fruit	Fruit eaten	4	MT-025
*Rumex abyssinicus* Jacq	Polygonaceae	Herb	Meqmoqo	Root	Root boiled in water and tea drunk	3	MT-191
*Rumex nervosus* Vahl	Polygonaceae	Shrub	Hahot	Leaf	Leaves eaten	7	MT-115, MT-162
				Stem	Stem chewed and juice swallowed		
*Sageretia thea* (Osbeck) M.C.Johnston	Rhamnaceae	Shrub	Agamqinchil	Fruit	Fruit eaten	9	MT-106, MT-170
*Smilax aspera* L	Similacaceae	Herb	Qalawadi (butign)	Fruit	Fruit eaten	1	MT-012
*Solanum nigrum* L	Solanaceae	Herb	Alamo	Fruit	Used as ingredient to make wot (stew) to be eaten with injera	2	MT-123
*Tamarindus indica* L	Fabaceae	Tree	Humer (roqa)	Fruit	Fruit eaten	2	MT-011
*Thymus serrulatus* Hochst. ex Benth	Lamiaceae	Herb	Toshne	Leaf	Leaf boiled in water and tea drunk	1	MT-016
*Ximenia americana* L	Olacaceae	Shrub	Muleo	Fruit	Fruit eaten	65	MT-004
*Ziziphus spina-christi* (L.) Desf	Rhamnaceae	Shrub	Qunqura	Fruit	Skin chewed and swallowed without stone	142	MT-002
-			Beso harestay	Root	Root eaten	3	
-			Mai atsgbi	Root	Root eaten after removing bark	11	
-			Baroda	Root	Root eaten after removing bark	1	
-			Katoita	Fruit	Fruit eaten without stone	1	
-			Kerbesha	Fruit	Fruit eaten	1	
-			Mugo mugoi	Fruit	Fruit eaten	1	
-			Tirumbule	Fruit	Fruit eaten	1	
-			Tirur	Fruit	Fruits ground and eaten after baking	1	

### Part consumed, taste, level of maturity at consumption and storage

**Fig. 2:**
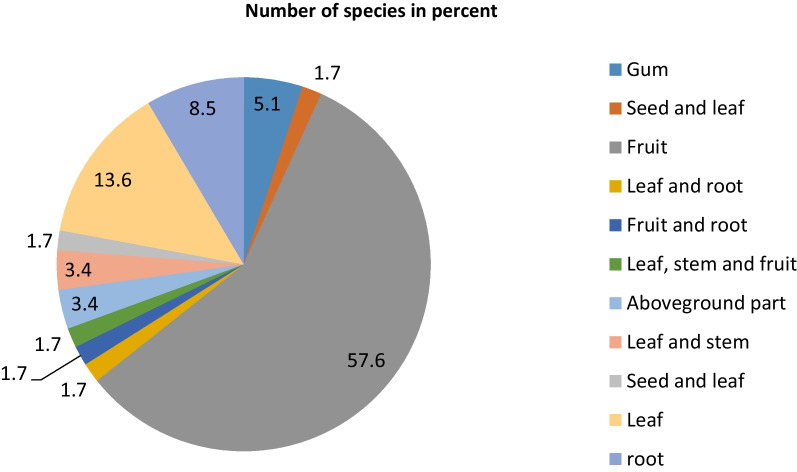
Proportions of parts of wild edible plants consumed in Raya-Azebo District

The majority (57.63%) of the WEPs in the study area were sought for their fruits, and few were harvested for their leaves (13.60%) and roots (8.5%) (Fig. 2). The edible fruits were claimed to have different tastes (sweet, sour, bitter) with the great majority having a sweet taste. The fruits were consumed when they got ripe, mostly characterized by color change from green to yellow, dark, purple or red. However, leafy vegetables were claimed to be consumed at their juvenile stage. There was little practice of storing WEPs in the area and thus the great majority of them were reported to be consumed immediately after harvesting while they were fresh.

### Preparation of edible parts and conditions of consumption

Most fruits were consumed raw by peeling off their skin (exocarp) and then chewing and swallowing with occasional spitting of seeds or stones (Table 1). On the other hand, the majority of the leafy vegetables were processed mainly by chopping, boiling and squeezing, and most frequently consumed with injera (pan-cake-like flatbread made of *Eragrostis tef* (Zucc.) Trotter).

The great majority of the wild edible plants in the study area were frequently harvested and consumed as supplementary/complementary foods at time of plenty or seasonal shortage of staple food. However, some (*Amaranthus hybridus* L., *Capsella bursa-pastoris* (L.) Medic.*, Cleome gynandra* L.*, Commiphora Africana* (A.Rich.) Engl.*, Cynanchum abyssinicum* Decne.*, Echidnopsis* sp.*, Huernia macrocarpa* (A.Rich.) Sprenger*, Eragrostis* sp., *Dobera glabra* (Forssk.) Pair.*, Pentarrbinum insipidum* E.Mey and *Rumex nervosus* Vahl) were only consumed at times of famine as reported by informants. Fruits were predominantly consumed by children, especially when herding animals in places that were far away from homesteads. On the other hand, leafy vegetables were usually harvested by women and prepared at home for household consumption.

### Season availability of wild edible plants

Analysis of data shows that the highest number of WEPs (49 species) in the study district were available for harvest in the autumn season (locally known as qewei), followed by those (37 species) that were harvested in the summer season (locally known as kiremti). The autumn season, which includes the months of September, October and November, comes after the long rainy summer season that includes the months of June, July and August. Twenty-six WEPs were consumed in the winter season (which includes the months of December, January and February), and 25 plants were consumed in the spring season which includes the months of March, April and May (Table 2). In terms of months, the highest number of WEPs was claimed to be consumed in September (43 species), followed by those consumed August (37 species), July (33 species), October (31 species) and November (31species). Some were consumed December (26 species), April (24 species), May (24 species), March (23 species), January (21 species), February (19 species) and June (18 species). The species *Acacia abyssinica* Hochst. ex Benth., *Acacia seyal* Del., *Balanites aegyptiaca*, *Carissa spinarum* L., *Cordia monoica* Roxb., *Cynanchum abyssinicum* Decne., *Grewia* sp., *Grewia villosa* Willd., *Huernia macrocarpa*, *Olea europaea* subsp. *cuspidata* (Wall. ex G.Don) cif. and *Rhus natalensis* Krauss, *Smilax aspera* L., and a plant locally known as katoita were reported to be available for harvest throughout the year.

**Table 2:**
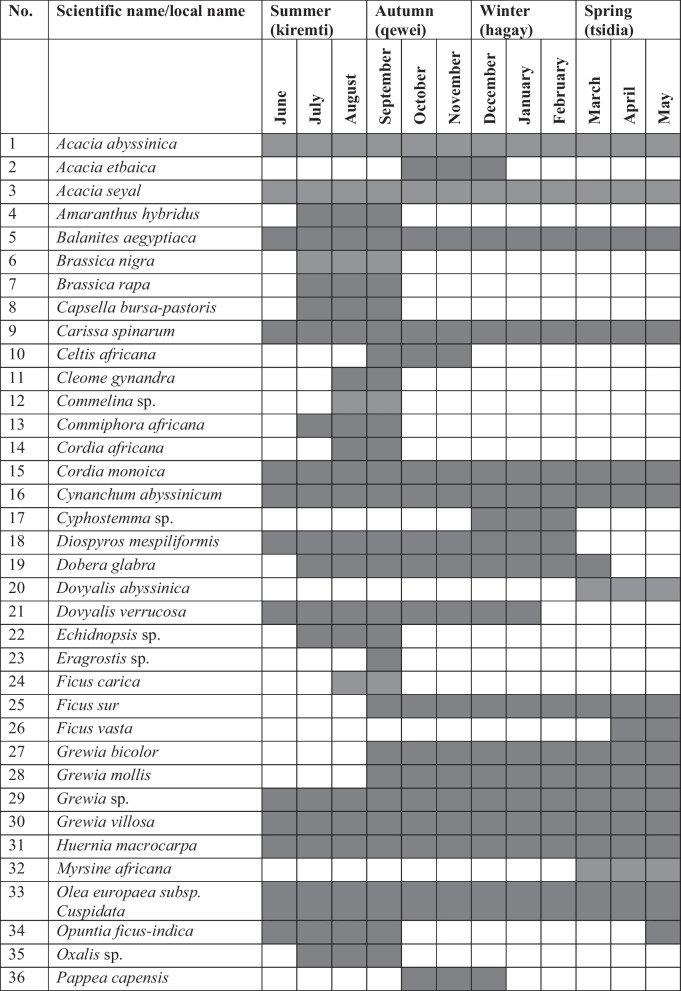

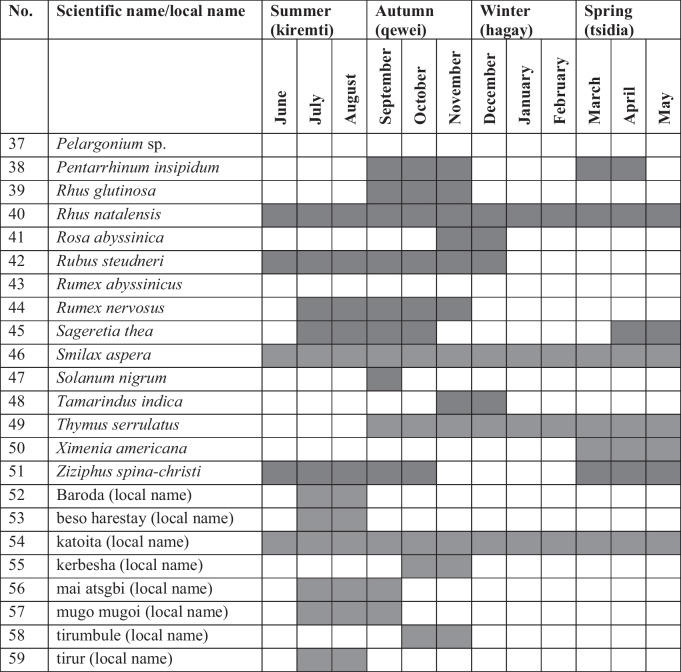
Seasons/months in which wild edible plants were harvested and consumed in Raya-Azebo District

Shaded areas show months that wild edible fruits were harvested and consumed

### Popular wild edible plants

Based on the number of informant citations, *Ziziphus spina-christi*, *Balanites aegyptiaca* and *Opuntia ficus-indica* were found to be the most popular WEPs in the district, cited by 142, 134 and 121 informants, respectively (Table 1). Other WEPs that were found popular include *Carissa spinarum*, *Cynanchum abyssinicum*, *Grewia* sp., *Ximenia Americana* L., *Grewia villosa*, *Cleome gynandra*, *Huernia macrocarpa*, *Cordia monoica*, *Ficus sur* and *Rhus natalensis*, reported by 97, 81, 68, 65, 45, 42, 29, 25, 20 and 20 informants, respectively (Table 1). A simple preference ranking exercise conducted on seven WEPs of the highest informant citations revealed *Opuntia ficus-indica*, *Ziziphus spina-christi* and *Balanites aegyptiaca* as the most preferred plants in the district (Table 3).

**Table 3:** Results of preference ranking exercise conducted on seven most cited wild edible plants in Raya-Azebo District

Plant name	Informants										Total score	Rank
	A	B	C	D	E	F	G	H	I	J		
*Opuntia ficus-indica*	7	7	7	7	7	7	7	7	7	7	70	1st
*Ziziphus spina-christi*	6	6	5	5	5	6	6	6	6	5	56	2nd
*Balanites aegyptiaca*	5	4	4	6	6	5	5	5	4	6	50	3rd
*Carissa spinarum*	4	5	6	4	3	3	4	4	5	4	42	4th
*Ximenia americana*	3	3	3	2	4	4	1	3	3	3	29	5th
*Grewia* sp.	2	2	2	3	2	2	3	2	2	2	22	6th
*Cynanchum abyssinicum*	1	1	1	1	1	1	2	1	1	1	11	7th

### Marketability

Interviews data showed that *Carissa spinarum*, *Sageretia thea* (Osbeck) M.C. Johnston, *Grewia villosa*, *Balanites aegyptiaca*, *Ficus vasta*, *Dovyalis abyssinica* (A.Rich.) Warb., *Ximenia americana*, *Opuntia ficus-indica*, *Ziziphus spina-christi*, *Ficus sur* and *Diospyros mespiliformis* Hochst. ex. A.DC. were sold at local markets for their food values*.* Whereas, market surveys witnessed the marketability of only four of the aforementioned plants that included *Opuntia ficus-indica, Ziziphus spina-christi, Ficus vasta, Ficus sur* and *Balanites aegyptiaca*.

### Habitat, availability and threats

Most of the WEPs consumed in the study area were harvested from farmlands and other disturbed habitats, roadsides, and woodlands. Very few were harvested from forested area. Nearly half of the reported WEPs were reported to have scarce occurrence in the area with the population of each plant continuing to decline from time to time. However, as interview reports indicated, very little effort has so far been made in the area to spare them from further devastation. The frequently mentioned threats of WEPs in the study area included agricultural expansion, recurrent drought and cutting of trees (for firewood purpose, house construction, making of farm tools, household utensils and fences). Ranking exercise conducted by informants revealed agricultural expansion and cutting of trees for firewood making as the main factors responsible for the depletion of WEPs in the district (Table 4). Of the claimed WEPs, *Ficus sur*, *Rhus natalensis*, *Ximenia americana* and *Ziziphus spina-christi* were reported to have rare occurrences in the study area.

**Table 4:** Results of preference ranking exercise to identify the main causes for the depletion of wild edible plants in Raya-Azebo District

Factor	Informants										Total score	Rank
	A	B	C	D	E	F	G	H	I	J		
Agricultural expansion	7	6	5	7	7	7	6	7	6	7	65	1
Recurrent drought	1	4	4	1	1	1	2	1	1	1	17	7
Use of trees as firewood	6	7	7	5	5	6	7	6	7	6	62	2
Use of trees for house construction	5	1	3	6	6	5	5	5	4	5	45	3
Tree-cutting for farm tools	2	2	2	3	2	4	3	3	3	2	26	5
Tree-cutting for house utensils	3	3	1	2	4	2	1	2	2	3	23	6
Tree-cutting for fencing	4	5	6	4	3	3	4	4	5	4	42	4

### Comparison of knowledge on wild edible plants among different social groups

Analysis of data collected revealed that there was a significant difference (*p* < 0*.*05) in the mean number of WEPs reported by literate and illiterate informants; the mean number WEPs reported by literate and illiterate informants were 6.69 ± 0.37and 5.45 ± 0.22, respectively. However, there was no significant difference in the number of WEPs reported by male (6.08 ± 0.23) and female (4.90 ± 0.43) informants, and those reported by informants above the age of 40 years and above (5.94 ± 0.23) and those who were below the age of 40 years (5.94 ± 0.52).

### Wild edible plants claimed to have medicinal values

Of the total recorded WEPs in the study district, 21 were reported to also have medicinal (nutraceutical) uses (Table 5). Of these, the plants *Balanites aegyptiaca* and *Acacia etbaica* Schweinf. had the highest informant agreement, reported by 17 and seven informants for their uses to manage anthrax and skin infections, respectively. *Balanites aegyptiaca* and *Acacia etbaica* also scored the highest rank order priority (ROP) values. *Balanites aegyptiaca* scored RPO value of 58.6 for its use to treat anthrax, and *Acacia etbaica* scored an RPO value of 43.8 for its use to manage skin infections (Table 6).

**Table 5:** Wild edible plants reported to have medicinal (nutraceutical) values in Raya-Azebo District

Scientific name/local name	Local disease name	English disease name	Part used	Method of preparation	Administration route
*Acacia abyssinica*	Qusli	Skin wound	Leaf	Pound leaves and dress wound with the paste	Dermal (local)
*Acacia etbaica*	Hebet	Skin wound	Leaf	Pound leaves and dress on the swollen part	Dermal (local)
	Neqersa	Skin wound	Leaf	Chew part and apply on the swollen part	Dermal (local)
	Shihur eid	Itchy skin of hands	Leaf	Chew and apply juice on itching skin	Dermal (local)
	Qusli	Skin wound	Leaf	Mix leaves of plant with that of *Cadia purpurea*, grind, add sour milk & apply on wound	Dermal (local)
	Anqer	Uvulitis	Leaf	Pound part, and apply paste on head after shaving	Dermal on head
	Boteta	Skin wound	Leaf	Pound leaves, and smear paste on the affected area	Dermal (local)
	Qusli	Skin wound	Leaf	Roast leaves on hot metal plate, crush, make paste in butter and apply on wound	Dermal (local)
	Qusli	Skin wound	Leaf	Rub leaves and tie them on the wound	Dermal (local)
*Balanites aegyptiaca*	Hibtet kisad	Wound on the neck	Root	Pound, add water, filter & sniff	Nasal
	Megerem	Anthrax	Root	Pound, filter and sniff small amount of the filtrate	Nasal
	Megerem	Anthrax	Root	Pound, filter and sniff small amount of the filtrate	Nasal
	Megerem	Anthrax	Root	Pound, add water, filter and sniff	Nasal
	Megerem	Anthrax	Leaf	Pound, dilute it in water, filter and sniff	Nasal
	Megerem	Anthrax	Stem bark	Pound part, and sniff	Nasal
	Megerem	Anthrax	Stem (bark)	Pound part, filter it, and add droplets into nostrils	Nasal
	Qusli	Skin wound	Root	Dry part, grind, add butter and smear paste on the wound	Dermal local on wound
	Megerem	Anthrax	Root	Pound part, mix in butter, heat it and smear paste on affected part	Dermal on affected part
	Habi	Taeniasis	Leaf	Pound part, sock it in water overnight, filter and drink one cup	Oral
	Megerem	Anthrax	Stem bark	Pound stem bark together with root of *Tribulus terrestris* and apply juice via the nostrils	Nasal
	Himam riesi, kebdi qurtset	Head ache, stomach ache	Root	Peel of the skin and eat the flesh and spit the seeds	Oral
	Megerem	Anthrax	Root	Pound part and apply few drops of the supernatant into the left nostril	Nasal
	Megerem	Anthrax	Stem (bark)	Pound bark, mix in water and take two cups of the supernatant orally or some drops nasally before meal	Oral, nasal
	Megerem	Anthrax	Root	Pound root, mix it in water, filter and drink filtrate	Oral
	Megerem	Anthrax	Stem (bark)	Pound part, mix it in water, filter and sniff filtrate via the left nostril	Nasal
	Megerem	Anthrax	Bark	Chop the internal part of the bark, mix it in small amount of water and drink; also apply some drops via the nostrils	Oral, nasal
	Megerem	Anthrax	Stem (bark)	Pound the bark, mix it in water, filter and apply little via mouth and nose	Oral, nasal
	Himam kebdi	Stomach problem	Fruit	Chew and swallow juice	Oral
	Himam kebdi	Abdominal problem	Fruit	Eat flesh and spit seeds	Oral
	Hibet	Swelling on the skin	Stem (bark)	Pound part after adding a liter of water, filter and drink a cup of the filtrate	Oral
	Uf shewa	Hepatitis	Stem (bark)	Pound part, mix it in water and drink juice	Oral
	Qurtset kebdi	Abdominal cramp	Fruit	Peel off the skin and eat flesh without the seeds	Oral
	Himam kebdi	Abdominal problem	Fruit	Peel off skin sock it in water filter and drink	Oral
	Megerem	Anthrax	Stem (bark)	Pound bark, add water and mix and apply few drops via nostrils	Nasal
	Teqmat	Diarrhea	Fruit	Remove skin, sock overnight in water, mix and drink	Oral
	Qurtset kebdi	Abdominal cramp	Root	Chew root and swallow the juice	Oral
	Megerem	Anthrax	Root	Pound, add little water, filter and sniff	Nasal
	Megerem	Anthrax	Root	Pound, add water, filter and sniff	Nasal
*Carissa spinarum*	Holeta (aso)	Malaria	Root	Boil it in water and drink and also sniff	Oral–nasal
	Michi	Febrile illness	Root	Boil part in water with root of *Withania somnifera* and leaves of bahir zaf and fumigate oneself	Nasal
	Michi	Febrile illness	Root	Boil part in water together with leaves of *Eucalyptus globulus* and *Ehretia cymosa* and fumigate oneself	Nasal
	Shegri	Crippling of legs	Root	Cut part and sock it in cold water for three days and wash body with it	Dermal
	Zebenegna	Mental illness	Root	Add part with roots of *Withania somnifera*, *Allium sativum*, *Lepidium sativum*, *Verbascum sinaiticum* and *Capparis tomentosa*, boil them in water and fumigate yourself with vapor	Nasal
	Ganen	Evil spirit	Root	Boil root of *Carrisa spinarum* in water with roots of *Bersama abyssinica* and *Justicia schimperiana* and fumigate oneself with steam	Nasal
	Egri liasir	Crippling of legs	Root	Sock root with roots of *Clerodendrum myricoides* for seven days and wash body with the supernatant	Dermal
	Holeta (aso)	Malaria	Root	Boil root in water and fumigate yourself with stem	Nasal
	Ide seb	Mental illness	Root	Pound part with fruit of *Citrus aurantifolia*, root of *Verbena officinalis*, root of *Solanum hastifolium*, root of *Capparis tomentosa* and root of *Corchorus* sp., sock in water for up to 7 days and wash with it	Dermal
	Ede seb	Mental illness	Root	Mix part with root of *Justicia schimperiana* and leaf or root of *Rumex nervosus*, add a liter of water and pound, and add juice of *Citrus lemon*, sugar and *Nigella sativa* and drink juice	Oral
	Michi	Unidentified febrile illness	Root	Boil root with that of *Withania somnifera* and fumigate yourself with steam	Nasal
	Qusli	Skin wound	Leaf	Pound leaves and smear paste on the wound	Dermal (local)
	Hibet	Swelling on the skin	Root	Dry stem bark, grind, mix in honey and dress swelling with paste	Dermal (local)
*Commiphora africana*	Himam kebdi	Abdominal problem	Resin	Chew resin and swallow juice	Oral
	Chebti	Gonorrhea	Stem (resin)	Chew resin and swallow juice	Oral
	Chebti	Gonorrhea	Root	Pound root, dilute it in water and drink juice	Oral
*Cordia monoica*	Zebenegna	Mental illness	Leaf	Add a number of leaves into hot coffee and drink; also massage legs with the socked leaves	Oral, dermal
*Dovyalis abyssinica*	Shihur	Itchy skin	Leaf	Sock parts in water, leave them over night and wash with liquid	Cutaneous
*Ficus carica*	Abiyi himam (lemtsi)	Vitiligo	Leaf	Pound leaves of the plant with bark of *Celtis africana* and *Acacia oerfota*, dry, mix in butter and smear paste on the skin	Dermal
*Ficus sur*	Uf shewa	Hepatitis	Fruit	Chop fruits, dry them, grind, mix powder in a water-full glass and drink	Oral
	Michi	Febrile illness	Fruit	Pound root with leaves of *Heliotropium cinerascens* and rub skin with paste; also put paste on hot metal and fumigate yourself with steam	Dermal, nasal
	Hibet	Swelling on the skin	Fruit	Dry, grind part together with pounded fresh leaves of *Conyza pyrrhopappa*, mix in honey and eat little amount and also smear paste on the skin	Oral
	Anker	Uvulitis	Fruit	Dry, grind together with dried seed of *Trigonella foenum-graecum*, mix in honey and apply on throat to ultimately swallow it	Oral
*Grewia* sp.	Boteta	Skin wound on hands and legs	Leaf	Chew leaves and dress paste on the affected areas	Dermal (local)
	Hibet	Swelling on the skin	Root	Chew root and dress juice on the swollen part	Dermal (local)
	Mich	Febrile illness	Root	Dry root bark, grind, put it on fire and fumigate yourself with smoke	Nasal, dermal
	Hawi semay	Herpes zoster	Leaf	Pound leaves and smear paste on the skin	Dermal
	Hibet	Swelling on the skin	Root (bark)	Pound part with root bark of *Grewia villosa* and smear on the swelling	Dermal (local)
*Grewia villosa*	Uf shewa	Hepatitis	Root (bark)	Pound, add water and drink juice	Oral
	Megerem	Anthrax	Leaf	Dry, grind and mix in honey and eat it	Oral
	Uf shewa	Hepatitis	Root (bark)	Chop down three finger-sized bark strips into smaller pieces, mix them in water and wash body below the neck with it	Dermal
	Uf shewa	Hepatitis	Root	Chop root and mix it in water and drink; also wash your face with it	Oral, dermal
	Hibet	Swelling on the skin	Root (bark)	Pound part with root bark of *Grewia sp.* And smear paste on the swelling	Dermal (local)
	Qurtset kebdi	Abdominal cramp	Stem (bark)	Pound fresh leaves of *Ziziphus spina-christi* with it the bark of *Grewia villosa*, add water, and then drink the liquid	Oral
	Hibet	Swelling on the skin	Leaf	Pound leaves after adding saliva, mix in honey and smear paste on the swollen part and dress it with a piece of cotton fabric	Dermal (local)
	Hibet	Skin infection	Leaf	Pound part and apply paste on wound	Dermal on affected part
*Myrsine africana*	Habi	Taeniasis	Fruit	Grind part, mix it in water and drink one glass on empty stomach	Oral
	Habi	Taeniasis	Fruit	Collect fruit and eat a hand-full of it	Oral
*Olea europaea* subsp. *Cuspidata*	Qitign	Syphilis	Root/stem	Burn and fumigate yourself with smoke	Body bath
	Bambule	Lymphogranuloma venereum	Root/stem	Burn and fumigate yourself with smoke	Body bath
	Kurtimat	Muscle ache	Root/stem	Burn and fumigate yourself with smoke	Body bath
	Holeta/aso	Malaria	Root/stem	Burn and fumigate yourself with smoke	Body bath
	Seal	Cough	Root/stem	Burn and fumigate yourself with smoke	Body bath
	Himam sini	Tooth ache	Leaf	Chew leaves and swallow juice to ease pain	Oral
	Holeta (aso)	Malaria	Stem	Put stem with stem of *Kleinia odora* on fire and fumigate oneself with smoke	Nasal
	Teqmat	Diarrhrea	Leaf	Pound part with root of *Solanum incanum* after adding a cup of water, filter and drink juice	Oral
*Opuntia ficus-indica*	Qusli	Skin wound	Stem (cladode)	Pound part and dress the wound with paste	Dermal (local)
	Qusli	Skin wound	Stem (cladode)	Cut cladode and apply jelly on the wound	Dermal on wound
*Pentarrbinum insipidum*	Anqer (ahniq)	Uvulitis	Root	The mother chew root and spit juice into the mouth of her child	Oral
*Rubus steudneri*	Wosfat, ameba	Ascariasis, amoebiasis	Fruit	Peel off skin and eat flesh	Oral
*Rumex nervosus*	Enewishin	Measles	Leaf/root	Pound, mix it with *Citrus aurantifolia* juice and apply on the skin	Cutaneous
	Shihur	Itchy skin	Leaf	Sock parts overnight in water and wash with liquid	Cutaneous
	Ede seb	Mental illness	Leaf, root	Mix parts with root of *Carissa spinarum* and root of *Justicia schimperiana*, add a liter of water and pound, and add *Citrus aurantifolia* juice, sugar and *Nigella sativa* and drink juice	Oral
*Ximenia americana*	Qusli	Skin wound	Stem bark	Grind the bark after drying using sun heat and sprinkle powder on the wound	Dermal (on the wound)
	Qusli	Skin wound	Stem (bark)	Pound fresh leaves of *Heliotropium cinerascens*, mix it in butter and dress the wound with paste; then add leaves of *Olea europaea* subsp. *Cuspidata* and apply paste on the wound	Dermal (local)
*Ziziphus spina-christi*	Qusli	Skin wound	Root (bark)	Pound part, add butter and apply on affected part	Dermal on affected part
	Forefor	Tinea capitis	Leaf	Pound leaves after adding some water and smear paste on the head	
	Himam kebdi	Abdominal problem	Fruit	Eat the skin of the plant	Oral
	Qurtset kebdi	Abdominal cramp	Leaf	Pound fresh leaves, add water, filter and drink the liquid	Oral
Tirumbila (local name)	Hibet	Swelling on the skin	Leaf	Pound leaves and dress affected part with paste	Dermal (local)
Titi (local name)	Efni	Joint swelling	Leaf	Dry, grind, mix it in butter and apply paste on swollen part	Local on swollen part

**Table 6:** Rank order priority and fidelity level values of medicinal plants reported by three or more informants against a given ailment in Raya-Azebo District

Species name	Ailment	IP	IU	FL (%) value	RPL	ROP
*Acacia etbaica*	Skin infections	7	8	87.5	0.5	43.8
*Balanites aegyptiaca*	Wound on the neck	3	29	10.3	1.0	10.3
	Anthrax	17	29	58.6	1.0	58.6
	Taeniasis	8	29	27.6	1.0	27.6
*Carissa spinarum*	Mich (febrile illness)	3	13	23.1	0.9	20.8
	Mental illness	4	13	30.8	0.9	30.1
*Grewia sp.*	Skin wound on hands and legs	3	5	60.0	0.3	18.0
*Grewia villosa*	Hepatitis	3	8	37.5	0.5	18.8
	Swelling on the skin	3	8	37.5	0.5	18.8

*IP *number of informants who reported the utilization of medicinal plants against a specific ailment, *IU* number of informants who mentioned the same plant against any ailment, *FL* fidelity level, *RPL* relative popularity level, *ROP* rank order priority

## Discussion

Results of the current study demonstrates that there is a wide use of wild edible as supplementary/complementary foods and nutraceuticals in Raya-Azebo District of the Tigray Region as revealed by the high diversity of the reported plant species. Relatively higher number of WEPs (59 species) was recorded from the study District as compared with those reported from other districts of the same region by Girmay et al. in Asgede Tsimbla, Tahtay Koraro and Medebay Zana districts (41 spp.) [39], Adhena in Raya Alamata District (37 spp.) [30], and Habtu in Wukro Kilte Awulaelo District (30 spp.) [31]. The wide use of WEPs in the district could be attributed to their good nutritional value as well as to the often-poor harvest of cultivated crops in the district mainly due to recurrent drought occurring in that part of the country [40, 41]. Based on literature survey, all the WEPs identified to a species level, except three (*Smilax aspera*, *Cynanchum abyssinicum* and *Pentarrbinum insipidum*), were also found to be consumed elsewhere in the country, which may be related to their better preference and/or wide occurrence in different agro-ecological zones of the country.

The fact that the families Asclepiadaceae and Fabaceae and Tiliaceae contributed a relatively higher number of wild edible species could be due to a combination of factors that, among others, may include their species diversity in Ethiopia and/or better nutritional value. Fabaceae is one of the few dominant dicotyledonous families in Ethiopia contributing 486 species [42]. This family is also rich in species that have high protein content [43]. The other two families, Asclepiadaceae and Tiliaceae, also have relatively fair diversity in the country, represented by170 [44] and 47 [45] species, respectively. Studies conducted in other parts of the country also show the common use of wild edible species belonging to the aforementioned three families [14, 17, 20, 22–24, 27, 30, 46–59]. Most WEPs in the study district were found to be shrubby species, which may demonstrate the better availability of the same for harvest throughout the year. Studies carried out elsewhere in the country also reported the common use of wild shrubby plants as a source of food [14, 20, 22, 27, 39, 48–52, 54, 58–60].

Most of the WEPs in the district were sought for their fruits, which could be due to rich nutritional content and good taste of fruits as also claimed by informants involved in the study. Many other studies conducted elsewhere in the country also witnessed the dominance of wild edible fruits [17, 19–24, 27, 30, 31, 39, 46, 48–55, 58–72].

The fact that there was little practice of harvesting and storing WEPs in the study district for later consumption may be attributed to the perishable nature of the consumed parts, especially the fruits and leaves, which were reported to be popular. Studies conducted elsewhere in Ethiopia also reported the perishability of wild fruits and leaves [62, 71], indicating their inconvenience for long-term storage. The common consumption of raw wild edible fruits may be taken as an effort to reduce the loss of nutritional values caused by boiling. Reports of similar studies conducted elsewhere in the country also showed the wide consumption of raw fruits [20, 22, 30, 31, 39, 47–50, 52, 54, 55, 58, 65, 67–69].

The majority of the WEPs in the district were harvested and consumed during the summer and autumn seasons including June, July, August, September, October and November, and that may attributed to the fact that their edible parts (mostly fruits) abundantly ripen at that time of the year. Several studies conducted in different parts of the country also reported better harvest and consumption of WEPs in the aforementioned seasons [30, 39, 57–59, 64, 68, 73] during which people often face a critical shortage of food. The species *Acacia abyssinica*, *Acacia seyal*, *Balanites aegyptiaca*, *Carissa spinarum*, *Cordia monoica*, *Cynanchum abyssinicum*, *Grewia* sp., *Grewia villosa*, *Huernia macrocarpa*, *Olea europaea* subsp. *cuspidata* and *Rhus natalensis*, *Smilax aspera*, and a plant locally known as katoita were revealed to be harvested and consumed year-round because of the availability of their edible parts, although the yield each plant may differ from season to season.

*Ziziphus spina-christi*, *Balanites aegyptiaca* and *Opuntia ficus-indica* were revealed as the most popular and preferred plants in the district, which may be attributed to their good harvest, taste and nutritional value. The fact that the three plants served as a good source of financial income, as also noted during interviews and market surveys, could have also contributed to their popularity. These plants were also found popular elsewhere in the northern part of the country [30, 31, 39, 55, 56, 64]. Laboratory investigation conducted elsewhere demonstrated the richness of *Ziziphus spina-christi* in fiber, carbohydrate and different minerals [74, 75], *Balanites aegyptiaca* in protein, fiber and different minerals [74–76], and *Opuntia ficus-indica* in carbohydrate, fiber and vitamin C [77, 78]. Preference ranking exercise revealed agricultural expansion and cutting of trees for their use as firewood as the leading factors for the depletion of WEPs in the district, which is also the case in many other parts of the country [19–21, 23, 24, 29–31, 39, 50, 52, 55, 56, 64, 66].

Analysis of data revealed that literate people (those who read and write) had better knowledge of the use of WEPs plants as compared to illiterate ones (those who do not read and write), which was in contrast to results of some studies conducted elsewhere in the country where illiterate people are more knowledgeable than literate ones [39, 58–70]. Education of most of the literate people in the study area is linked to religious institutions (mostly Christianity) and that might have contributed to their better knowledge of WEPs. Some manuscripts belonging to Christianity in different parts of the world often provide information on useful plants including medicinal and wild edible plants [79, 80].

Of the WEPs reported to have medicinal (nutraceutical) values in the study district, *Balanites aegyptiaca* and *Acacia etbaica* scored the highest rank order priority (ROP) values, *Balanites aegyptiaca* for its use to treat anthrax and *Acacia etbaica* for its use to manage skin infections. Investigations conducted elsewhere in the country also revealed the use of *Acacia etbaica* against skin infection [81–83], and the use of *Balanites aegyptiaca* against anthrax [84, 85]. Furthermore, some investigations demonstrated the antibacterial properties of *Acacia etbaica* [86, 87] and *Balanites aegyptiaca* [88–90], which corroborate the local uses of the two plants against the aforementioned health problems.

## Conclusions

The current investigation demonstrated a wide use of WEPs in Raya-Azebo district as revealed by the high diversity of recorded plants (59 species), the majority of which were sought for their fruits. Most of the plants were consumed, as supplementary foods, and often by children. The highest number of WEPs was consumed in the autumn season, which includes the months of September, October and November from which September took the lead. The plants *Ziziphus spina-christi*, *Balanites aegyptiaca* and *Opuntia ficus-indica* were found to be the most preferred WEPs. Agricultural expansion and cutting of trees for firewood purpose were found to be the main conservation threats for WEPs. Of the total WEPs, 21 were reported to also have medicinal (nutraceutical) values. *Balanites aegyptiaca* and *Acacia etbaica* scored the highest rank order priority (ROP) values, the former for its use to treat anthrax and the later for its use to manage skin infections. In future evaluation of the nutritional value of the documented WEPs, priority should be given to those that were found popular in the study district. Likewise, priority should be given to nutraceutical plants that scored the highest ROP values in the investigation of pharmacological properties and phytochemical profiles. Furthermore, immediate attention should be given by concerned individuals and institutions in the country to manage (in situ and ex situ) wild edible and nutraceutical plants that were reported to have rare occurrences in the study District by involving the local community.

## Data Availability

Data related to this study were stored in a desktop computer available at Aklilu Lemma Institute of Pathobiology (ALIPB), Addis Ababa University (AAU). Readers may get access to the data through request made to ALIPB. Plant voucher specimens have been deposited at the mini-herbarium of Endod and Other Medicinal Plants Research Unit, ALIPB, AAU.

## Abbreviations

AAU: Addis Ababa University
ALIPB: Aklilu Lemma Institute of Pathobiology
ANOVA: Analysis of variance
FL: Fidelity level
ROP: Rank order priority
RPL: Relative popularity level
SEM: Standard error of the mean
SPSS: Statistical Package for Social Sciences
WEPs: Wild edible plants
